# Clinical utility of lipid ratios as potential predictors of metabolic syndrome among the elderly population: Birjand Longitudinal Aging Study (BLAS)

**DOI:** 10.1186/s12877-023-04040-8

**Published:** 2023-07-03

**Authors:** Farhad Saeedi, Elnaz Baqeri, Ali Bidokhti, Mitra Moodi, Farshad Sharifi, Seyed Mohammad Riahi

**Affiliations:** 1 Student Research Committee, Birjand University of Medical Sciences, Birjand, Iran; 2 Cardiovascular Diseases Research Center, Birjand University of Medical Sciences, Birjand, Iran; 3 Social Determinants of Health Research Center, Birjand University of Medical Sciences, Birjand, Iran; 4 Elderly Health Research Center, Endocrinology and Metabolism Population Sciences Institute, Tehran University of Medical Sciences, Tehran, Iran; 5 Cardiovascular Diseases Research Center, Department of Epidemiology and Biostatistics, School of Medicine, Birjand University of Medical Sciences, Birjand, Iran

**Keywords:** Metabolic syndrome, Lipids, Aged, Frail Elderly, Geriatrics

## Abstract

**Background:**

Elderly adults are at higher risk of developing metabolic syndrome (MetS). The present study aims to investigate the relationship between lipid ratios and MetS in the elderly population.

**Methods:**

This study was conducted on elderly population of Birjand during 2018–2019. The data of this study was driven from Birjand Longitudinal Aging Study (BLAS). The participants were selected based on multistage stratified cluster sampling. Patients were categorized into quartiles according to the lipid ratios (TG/HDL-C, LDL-C/HDL-C, non-HDL/HDL-C), and the relationship between lipid ratio quartiles and MetS was determined by Logistic Regression using Odds Ratio. Finally, the optimal cut-off for each lipid ratio in MetS diagnosis was calculated according to the Area Under the Curve (AUC).

**Results:**

This study included 1356 individuals, of whom 655 were men and 701 were women. In our study, the crude prevalence of MetS was 792 (58%), including 543 (77.5%) women and 249 (38%) men. Increasing trends were observed in quartiles of all lipid ratios for TC, LDL-C, TG, and DBP. TG/HDL was also the best lipid ratio to diagnose the MetS, based on NCEP ATP III criteria. One unit increased in level of TG/HDL resulted in 3.94 (OR: 3.94; 95%CI: 2.48–6.6) and 11.56 (OR: 11.56; 95%CI: 6.93–19.29) increasing risk of having MetS in quartile 3 and 4 compared to quartile 1, respectively. In men and women, the cutoff for TG/HDL was 3.5 and 3.0, respectively.

**Conclusions:**

Our results showed that the TG/HDL-C is superior to the LDL-C/HDL-C and the non-HDL /HDL-C to predict MetS among the elderly adults.

## Introduction

Metabolic Syndrome (MetS) is characterized by a combination of disorders including hyperglycemia, dyslipidemia, hypertension, and abdominal obesity and shows an increasing growth pattern during the recent decades [1, 2]. MetS prevalence is highly variable based on the MetS definition Criteria and the studied population ranging between < 10–84%; though, MetS prevalence is globally estimated to be nearly 20–25% in the adults [3]. The growing prevalence of MetS was confirmed by a population-based cohort study in Iran, rising from 35.6% to 2001 to 42.5% in 2013 [4]. Studies have shown the association between MetS and obesity, higher level of income, sedentary lifestyle, and urbanization [5–8]. Also, the studies have confirmed the higher risk of cognitive impairment, diabetes mellitus type 2, and chronic renal diseases in patients with MetS [9–13]. On the other hand, accumulating evidence demonstrates the increased rate of cardiovascular diseases, all-cause mortality, and diabetes mellitus cardiovascular mortality [14–16].

MetS early diagnosis and treatment effectively prevents the development of undesired complications; thus, clinically, it is beneficial to identify the proper criteria for accurate differentiation of patients with MetS and healthy individuals [17]. The NCEP ATP III, a commonly used MetS diagnosis criteria, requires waist circumference (WC) measurement [18], which is not usually performed in clinical settings, thereby postponing the MetS early diagnosis [19]. Recently, alternative lipid ratio indices such as low-density lipoprotein cholesterol (LDL-C)/ HDL-C, triglyceride (TG)/HDL-C, and non high-density lipoprotein cholesterol /high-density lipoprotein cholesterol (non-HDL /HDL-C) are suggested for Mets diagnosis [1, 19–22]. It seems that lipid ratios (using two lipid serum levels) are clinically more practical compared with individual lipid levels in MetS diagnosis [19, 23–25], though limited studies have been conducted on the accuracy of this approach in MetS diagnosis on specific populations [19, 26–28].

The rate of MetS varies in different age groups due to physiologic factors, e.g., postmenopausal women are at higher risk of MetS due to hormonal alterations [29]; also, the prevalence of MetS among the elderly is increasing worldwide due to the age-related physiologic changes and the elderly are at higher risk of abdominal obesity and MetS [30]. Sex difference in the prevalence of MetS is clinically important and may result from sex hormone effects, leading to insulin resistance, obesity, and hypertension [31–33]. Particularly in a recent study, it’s been shown that higher level of testosterone and sex hormone binding globulin were associated with increased insulin sensitivity and decreased risk of MetS development in elderly male [34].

MetS may lead to several diseases and complications that highly affect the individuals’ health status. On the other hand, no studies have been conducted on the diagnostic accuracy of lipid ratios, especially in the elderly. The present study aims to investigate the relationship between lipid ratios and MetS in the male and female elderly population in Birjand.

## Methods and materials

### Study population and data collection

This analytic epidemiologic study was conducted on the elderly population of Birjand, East of Iran during 2018–2019. The study participants were selected using random cluster sampling based on multistage stratified cluster sampling. The details about study design of Birjand Longitudinal Aging Study (BLAS) were discussed elsewhere [35]. Briefly, the dataset of this study was obtained from Sib electronic, which is a national health records dataset, and hand registry systems. This study includes urban participants from Birjand county.

Inclusion and exclusion criteria.

People who were 60 years old or older and were able to participate study, meaning that visiting the research center and complete the questionare and physical exam and laboratory data, were include. The people who were unable to participte study including those who were bedridden or had sever cognition disorder were excluded. Moreover, those elderly adults who were lost to follow up were excluded. The people who were not interested in participating study were excluded.

### Covariate

After proposal approval and obtaining the research ethics code, data was obtained from the Social Determinants of Health (SDH) research center regarding the comprehensive plan for the elderly. Clinical and laboratory measurmenents were discussed elsewhere [35]. The primary outcome variable was MetS, defined according to NCP/ATP III definition [18].

We defined MetS in the presence of at least three of the following criteria based on NCP/ATP III definition: (1) waist circumference ≥ 102 cm in men and ≥ 88 cm in women, (2) triglyceride ≥ 150 mg/dL or on drug treatment for dyslipidemia, (3) HDL-C < 40 mg/dL in men and < 50 mg/dL in women, (4) blood pressure ≥ 130 mm Hg systolic blood pressure or ≥ 85 mm Hg diastolic blood pressure or on antihypertensive treatment, (5) fasting blood glucose ≥ 100 mg/dL or on antidiabetic treatment [36] .

Independent variables in this study included different lipid ratios (TG/HDL, LDL/HDL, and non-HDL/HDL). The confounding variables were BMI, smoking, hypertension, and diabetes mellitus. Patients were categorized into quartiles according to the lipid ratios, and the relationship between lipid ratio quartiles and MetS was determined by Logistic Regression using Odds Ratio.

### Lipid ratio calculation

Lipid ratios were calculated by dividing the TG, LDL-C, and TC values by HDL-C values. The unites for all the lipid variables were mg/dl. Non-HDL is calculated as TC minus HDL, so that the ratio of non-HDL/HDL equals to (TC/HDL)-1.

### Statistical analysis

In this study, qualitative data are reported as frequency and percentage, and quantitative variables were reported as mean and standard deviation or median (first and third quartiles). The standard indices were calculated based on the WHO standard world population 2000 [37]. Chi-square or Fisher’s exact test was used to compare qualitative variables between patients with and without MetS. Data Normality was confirmed by the Kolmogorov–Smirnov test and P-P plot. T-test or MannWhitney test was used to compare the means of the quantitative variable between patients with and without MetS. Logistic regression was used to determine the relationships between lipid ratios and MetS. Lipid ratios were divided in 4 groups (quartile). The first quartile was used as a reference group against which the remaining quartiles were compared. Area Under the Curve (AUC) and Hosmer–Lemeshow test was used to goodness of fit the statistical models. ROC curve analysis was used to determine the optimal cut-off points for each lipid ratio. Youden index was calculated as follows: sensitivity (%) + specificity (%) – 100 [38]. Statistical analysis was performed using SPSS version 22, and a P-value below 0.05 was considered statistically significant.

## Results

Characteristics are shown in Table 1. This study included 1356 individuals, of whom 655 were men and 701 were women. In our study, 792 (58%) participants were diagnosed with MetS, including 543 (77.5%) women and 249 (38%) men. The mean age of patients with MetS was 69.2 ± 7.01 years. BMI, WC, FBS, TG, SBP, and DBP were significantly higher among the MetS group. However, TC was not significantly different between the non-MetS and MetS groups. Moreover, HDL-C and LDL-C were significantly lower in the MetS group. In contrast to LDL/HDL, TG/HDL was significantly higher in the MetS group compared to the non-MetS group. non-HDL/HDL was significantly higher among the total population and women among MetS groups.

**Table 1:** Baseline characteristics of individuals with and without MetS

Variables	Total (1356)			Men (655)			Women (701)		
	METS -	METS+	PVALUE	METS-	METS+	PVALUE	METS-	METS+	PVALUE
	564(42%)	792(58%)		406 (62%)	249(38%)		158(23%)	543(77%)	
**Age**	70.4(8.1)	69.2(7.01)	0.005	71.1(8.2)	70.0(7.0)	0.046	68.6(70.6)	69.0(7.0)	0.643
**BMI**	24.0(4.7)	28.2(5.0)	< 0.001	23.7(4.7	27.1(3.7)	< 0.001	24.7(4.7)	28.7(5.4)	< 0.001
**WC**	88.3(10.6)	100.2(10.0)	< 0.001	88.3(10.6)	99.8(9.6)	< 0.001	88.4(10.4)	100.5(10.3)	< 0.001
**FBS**	97.0(26.3)	119.8(40.3)	< 0.001	98.4(29.2)	125.5(37.9)	< 0.001	93.5(16.0)	117.2(41.3)	< 0.001
**TG**	129.4(46.5)	171.0(71.9)	< 0.001	129.8(49.2)	179.4(78.5)	< 0.001	128.4(39.0)	167.0(68.3)	< 0.001
**SBP**	127.4(20.7)	134.9(20.2)	< 0.001	131.2(20.6)	142.3(18.5)	< 0.001	117.6(17.5)	131.5(20.1)	< 0.001
**DBP**	76.3(11.2)	79.6(11.3)	< 0.001	77.2(11.4	82.0(11.5)	< 0.001	74.1(10.4)	78.6(11.1)	< 0.001
**TC**	196.8(38.4)	197.9(43.2)	0.605	192.89(38.8)	189.6(47.8)	0.369	207.0(35.3)	201.8(40.3)	0.137
**HDL-C**	44.8(5.0)	43.0(5.1)	< 0.001	44.1(4.4)	41.8(5.3)	< 0.001	46.5(5.9)	43.5(4.9)	< 0.001
**LDL-C**	125.7(34.4)	121 (37.3)	0.017	122.5(34.5)	113.4(39.7)	0.003	133.9(33.0)	124.4(35.7)	0.003
**Non-HDL/HDL**	4.4(0.9)	4.7 (1.1)	< 0.001	4.4(1.0)	4.5(1.3)	0.058	4.5(0.9)	4.7(1.1)	0.038
**TG/HDL**	3.0(1.2)	4.1(2.0)	< 0.001	3.0(1.3)	4.4(2.3)	< 0.001	2.8(1.0)	4.0(1.8)	< 0.001
**LDL/HDL**	2.8(0.8)	2.8(0.9)	0.817	2.8(0.8)	2.7(1.0)	0.422	2.9(0.8)	2.9(5.9)	0.761

Biomarkers and variables of MetS in different quartiles of lipid ratios are shown in Table 2. While HDL-C showed a decreasing trend, TC, LDL-C, TG, and DBP significantly raised across the quartiles of all lipid ratios (P-value < 0.001). Moreover, our results showed an increasing trend in WC and BMI across the quartiles of non-HDL/HDL and TG/HDL (P-value < 0.001). However, FBS and WHR significantly increased across the TG/HDL and LDL/HDL quartiles (P-value < 0.001).

**Table 2:** Biomarkers and components of MetS in quartiles of lipid ratios

Quartiles of non-HDLC/HDL-C Ratio	Q1 (< 2.81)		Q2 (2.81–3.47)		Q3 (3.47–4.18)		Q4 (> 4.18)		P-trend
N = 1356	339 (25%)		336(24.81%)		341 (25.2%)		339 (25%)		
	Mean	SD	Mean	SD	Mean	SD	Mean	SD	
WC (cm)	94.3	11.9	95.0	11.5	96.8	12.0	95.1	11.7	< 0.001
BMI	26.0	5.7	26.1	5.0	27.1	5.8	26.4	4.7	< 0.001
TC (mg/dl)	149.4	22.0	187.2	19.9	212.5	23.2	240.6	29.6	< 0.001
HDL-c (mg/dl)	45.0	4.5	45.0	4.5	44.2	4.6	40.7	5.6	< 0.001
LDL-c (mg/dl)	81.5	17.5	114.0	18.9	135.8	22.6	160.1	26.9	< 0.001
TG (mg/dl)	122.1	41.4	141.9	48.1	159.0	59.7	191.4	84.6	< 0.001
SBP (mm Hg)	76.7	11.6	77.2	10.9	78.8	11.4	80.1	11.3	< 0.001
DBP (mm Hg)	132.4	20.6	130.3	20.6	132.3	21.7	132.0	20.1	0.506
Met-total day	2.9	4.5	3.2	4.5	3.2	5.3	3.6	5.6	0.361
WHR (cm)	1.0	0.1	1.0	0.1	1.0	0.1	0.9	0.1	0.179
Hip (cm)	98.4	10.7	99.1	10.3	101	11.1	100.5	11.3	< 0.001
FBS (mg/dl)	111.3	36.5	110.7	37.1	109.6	35.1	109.6	39.1	0.902
**Quartiles of TG/HDL-C Ratio**	**Q1 (< 2.47)**		**Q2 (2.47–3.21)**		**Q3(3.21–4.25)**		**Q4 (> 4.25)**		**P-trend**
**N = 1356**	**338 (24.9%)**		**343(25.3%)**		**340 (25.1%)**		**335 (24.7%)**		
	**Mean**	**SD**	**Mean**	**SD**	**Mean**	**SD**	**Mean**	**SD**	
WC (cm)	91.5	12.8	94.2	18.6	97.1	11.2	98.5	10.3	< 0.001
BMI	24.7	5.0	26.4	5.3	27.2	5.6	27.4	4.9	< 0.001
TC (mg/dl)	178.5	37.5	198.1	38.6	197.8	40.2	215.6	40.2	< 0.001
HDL-c (mg/dl)	45.6	4.8	45.1	4.2	43.0	4.6	41.2	5.6	< 0.001
LDL-c (mg/dl)	114.1	33.6	125.5	36.3	123.7	37.4	128.4	36.0	< 0.001
TG (mg/dl)	89.9	14.3	129.4	13.8	158.3	21.0	238.1	69.4	< 0.001
SBP (mm Hg)	76.0	11.0	78.8	11.9	78.1	10.8	80.0	11.6	< 0.001
DBP (mm Hg)	130.4	21.8	132.4	21.3	130.7	19.7	133.6	20.0	0.142
Met-total day	3.0	4.3	3.7	6.2	3.0	4.0	3.1	5.2	0.273
WHR (cm)	0.9	0.1	0.9	0.1	1.0	0.1	1.0	0.1	< 0.001
Hip (cm)	96.4	10.7	99.5	10.6	101.2	11.0	101.9	10.5	< 0.001
FBS (mg/dl)	101.5	24.7	108.2	38.4	110.7	31.5	120.9	47.0	< 0.001
**Quartiles of LDL/HDL ratio**	**Q1(< 2.15)**		**Q2 (2.15–2.80)**		**Q3 (2.80–3.42)**		**Q4 (> 3.42)**		**P-trend**
**N = 1356**	**338 (24.9%)**		**340 (25.1%)**		**338 (24.9%)**		**340 (25.1%)**		
	**Mean**	**SD**	**Mean**	**SD**	**Mean**	**SD**	**Mean**	**SD**	
WC (cm)	95.4	11.7	94.9	11.5	96.5	11.7	94.5	12.3	0.150
BMI	26.4	5.7	26.3	5.3	26.7	5.3	26.4	5.0	0.823
TC (mg/dl)	151.7	24.4	187.1	23	210.6	25.7	240.4	28.3	< 0.001
HDL-c (mg/dl)	44.7	4.5	44.8	4.9	44.1	4.7	41.3	5.5	< 0.001
LDL-c (mg/dl)	78.5	14.4	111.7	15.2	136.3	16.4	165	22.1	< 0.001
TG (mg/dl)	140.0	62.9	149.1	59.6	151.9	59.7	173.5	75.4	< 0.001
SBP (mm Hg)	133.2	20.1	130.3	21.9	132.4	21.2	131.2	19.7	0.259
DBP (mm Hg)	77.2	11.7	77.0	11.5	78.9	11.5	79.8	10.6	< 0.001
Met-total day	2.9	4.7	3.0	3.9	3.5	5.6	3.5	5.6	0.207
WHR (cm)	1.0	0.1	1.0	0.1	1.0	0.1	0.9	0.1	0.030
Hip (cm)	99.2	10.6	99.2	10.7	100.7	10.6	100.0	11.6	0.222
FBS (mg/dl)	116.9	42.3	108.2	33.6	107.9	31.8	108.1	38.4	0.002

The probability of developing MetS was not statistically significant across the quartiles of all lipid ratios (Table 3). However, TG/HDL was the best predictable lipid ratio for the incidence of MetS. It was shown that a 1 unit increased in level of TG/HDL resulted in 3.94 (OR: 3.94; 95%CI: 2.48–6.6) and 11.56 (OR: 11.56; 95%CI: 6.93–19.29) increasing risk of having MetS in quartile 3 and 4 compared to quartile 1, respectively. This increasing trend was also observed in both men and women groups.

**Table 3:** The relation between chance of MetS development and lipid ratios

		OR	(95%CI)	OR	(95%CI)	OR	(95%CI)	AUC	HLT
**Quartiles of non-HDL/HDL-C Ratio**									
	**Q1 (< 2.81)**	**Q2 (2.81–3.47)**		**Q3 (3.47–4.18)**		**Q4 (> 4.18)**			
**Total**	Reference	1.11	(0.72–1.71)	1.56	(1.01–2.41)	2.98	(1.90–4.69)	0.90 (0.88–0.91)	0.854
**Men**	Reference	1.21	(0.66–2.19)	1.82	(1.00-3.31)	3.14	(1.79–5.28)	0.88 (0.85–0.90)	0.475
**women**	Reference	0.93	(0.48–1.80)	1.25	(0.65–2.40)	2.5	(1.26–4.94)	0.88 (0.85–0.90)	0.926
**Quartiles of TG/HDL-C Ratio**									
	**Q1 (< 2.47)**	**Q2 (2.47–3.21)**		**Q3(3.21–4.25)**		**Q4 (> 4.25)**			
**Total**	Reference	1.11	(0.67–1.60)	3.94	(2.48–6.6)	11.56	(6.93–19.29)	0.92 (0.91–0.93)	0.388
**Men**	Reference	1.21	(0.45–1.78)	4.45	(2.33–8.50)	12.19	(6.16–24.12)	0.90 (0.88–0.93)	0.198
**women**	Reference	0.93	(0.62–2.08)	3.43	(1.74–6.76)	9.36	(4.27–20.48)	0.90 (0.88–0.92)	0.556
**Quartiles of LDL/HDL-C Ratio**									
	**Q1 (< 2.15)**	**Q2 (2.15–2.80)**		**Q3 (2.80–3.42)**		**Q4 (> 3.42)**			
**Total**	Reference	1.03	(0.67–1.60)	1.3	(0.84-2.00)	1.77	(1.12–2.77)	0.89 (0.88–0.91)	0.884
**Men**	Reference	1.01	(0.56–1.81)	1.12	(0.63-2.00)	1.67	(0.91–3.03)	0.87 (0.84–0.90)	0.408
**women**	Reference	1.12	(0.56–2.22)	1.55	(0.78–3.08)	1.84	(0.91–3.70)	0.87 (0.84–0.90)	0.855

**Fig. 1:**
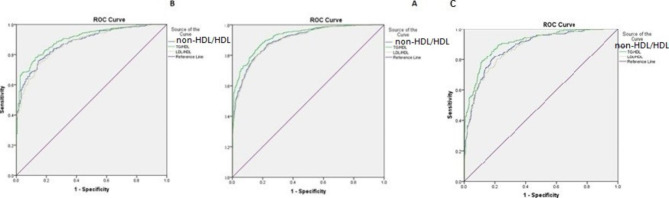
ROC curve for the value of lipid ratios for prediction of MetS among general population **(A)**, men **(B)**, and women **(C)**

**Fig. 2:**
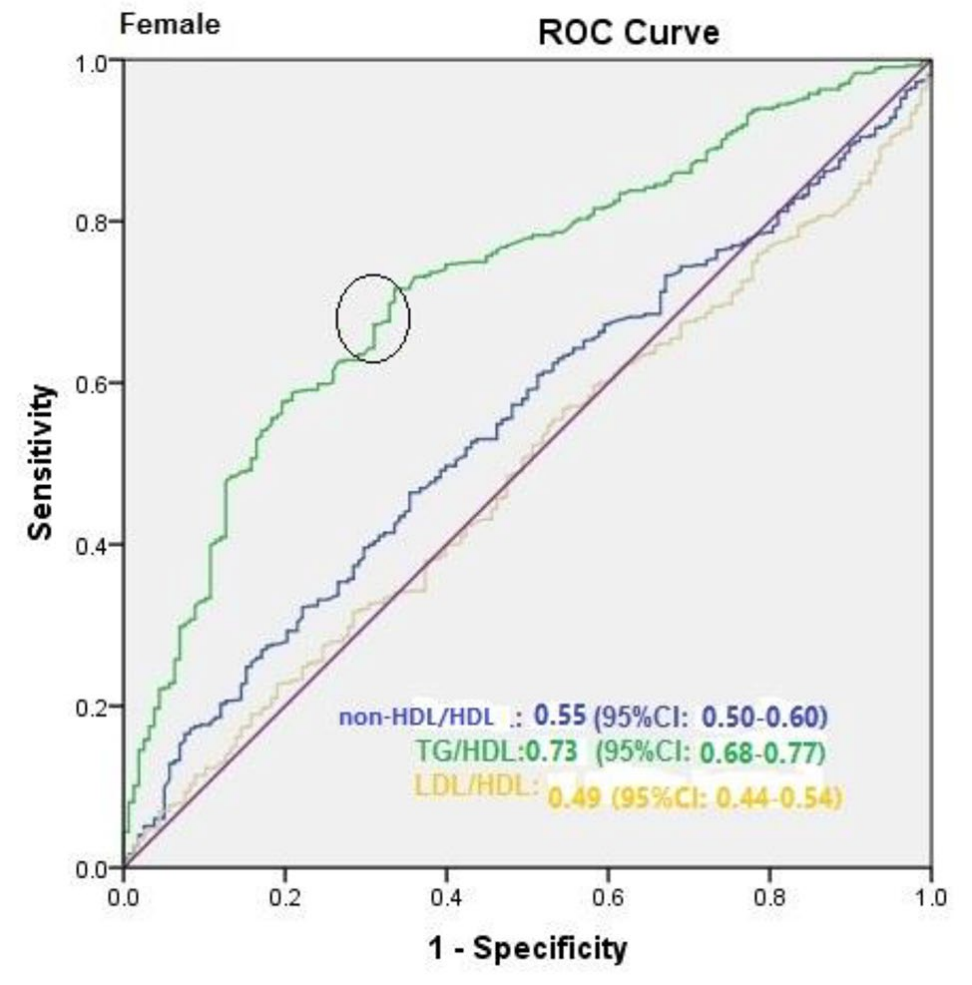
ROC models of asscociation between lipid ratios and MetS in women after excluding confounding variables

**Fig. 3:**
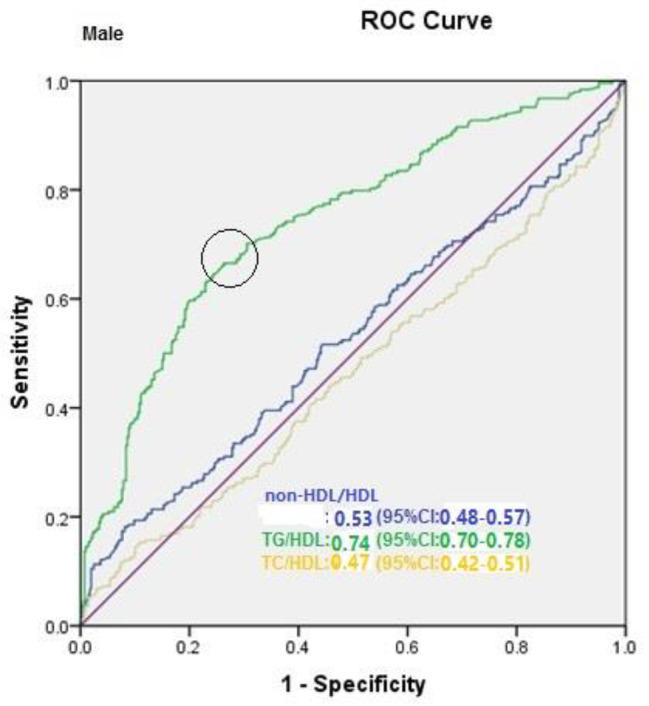
ROC models of asscociation between lipid ratios and MetS in men after excluding confounding variables

The AUC of TG/HDL was greater than other lipid ratios, suggesting that TG/HDL is a more reliable MetS predictor (Fig. 1). Including the confounding variables, the AUC of LDL/HDL, TG/HDL, and non-HDL/HDL for diagnosis of MetS were 0.89 (0.88–0.91), 0.92 (0.91 to 0.93), and 0.90 (0.88 to 0.91), respectively (Fig. 1A). Similar patterns were found for men and women, with an approximately same AUC for both groups (Fig. 1B C). The AUC of LDL/HDL, TG/HDL, and non-HDL/HDL for diagnosis of MetS in men were 0.87 (0.84–0.90), 0.90 (0.88 to 0.93), and 0.88 (0.85 to 0.90), respectively. The AUC of LDL/HDL, TG/HDL, and non-HDL/HDL for diagnosis of MetS in women were 0.87 (0.84–0.90), 0.90 (0.88 to 0.92), and 0.88 (0.85 to 0.90), respectively. Moreover, the ROC models of asscociation between lipid ratios and MetS in women and men, after excluding confounding variables, were shown in Figs. 2 and 3. In women, the AUC for non-HDL/HDL, TG/HDL, LDL/HDL were 0.55 (95%CI: 0.50–0.60), 0.73 (95%CI: 0.68–0.77), and 0.49 (95%CI: 0.44–0.54), respectively. The P-value of ROC model for non-HDL/HDL, TG/HDL, LDL/HDL after excluding confounding variables were 0.05, 0.001, and 0.69 in women, respectively. In men, the AUC for non-HDL/HDL, TG/HDL, LDL/HDL were 0.53 (95%CI: 0.48–0.57), 0.74 (95%CI: 0.70–0.78), and 0.47 (95%CI: 0.42–0.51), respectively. The P-value of ROC model for non-HDL/HDL, TG/HDL, LDL/HDL HDL after excluding confounding variables were 0.26, 0.001, and 0.13 in men, respectively. In men and women, the cutoff for TG/HDL was 3.5 and 3.0, respectively. Youden index of TG/HDL for men and women was 40 and 39, respectively. The ROC curve of TC/HDL would overlap non-HDL/HDL. The quartiles of non-HDL/HDL would be as the same as TC/HDL. So there would be no differences in ROC model and quartiles of non-HDL/HDL compared to TC/HDL.

## Discussion

This study aimed to investigate the association of MetS with lipid ratios among the elderly. It was found that BMI, WC, SBP, DBP, WHR were significantly higher in the MetS group compared to the non-MetS group. Moreover, increasing trends were observed in quartiles of all lipid ratios for TC, LDL-C, TG, and DBP. TG/HDL was also the best lipid ratio to predict the diagnosis of MetS, based on NCEP ATP III criteria.

Sex differences in metabolic syndrome prevalence might be due to abdominal obesity, hormone modulation, and glucose metabolism [39]. Moreover, the inflammatory process responsible for MetS might be sex-specific. A recent study showed increased level of inteleukin-6, cytokine, and leptin in men as well as decreased level of adiponectin in women [40].

Dyslipidemia has an important role in the pathophysiology of MetS. Abdominal obesity might give rise to hypertrophic adipocytes, inducing insulin resistance. Furthermore, decreased retention of free fatty acid in adipocytes, increased production of very low density lipoprotein (VLDL) apo B-100, decreased catabolism of apo B comprising particles, and increased catabolism of HDL-apo A-I may all result in increased levels of free fatty acid, VLDL, and decreased levels of HDL-C in plasma [41]. Cholesterol is transported by various lipoproteins in plasma. The main carrier of cholesterol from liver is LDL, by which cholesterol deposits in intima layers of arteris, resulting in formation of atherogenic plaques [42]. However, HDL transports cholesterol to liver and decreases the risk of atherosclerorsis [42]. The plasma level of cholesterol increases with aging, leading to increased risk of cardiovascular disease [43, 44].

MetS might give rise to microvascular damage, leading to endothelial dysfunction, increased vascular resistance, atherosclerosis,and hypertension [45]. In addition, the level of adiponectin decreases in patients with MetS,which might eventually result in coronary heart disease [46, 47]. A growing body of studies showed that MetS is prevalent among the elderly in Iran [48–50]. Although there is some conflicting evidence regarding MetS mortality in the elderly [51], it is recommended that they undergo MetS screening to lower the probability of developing severe complications as well as the cost to the public health system [52].

It has been shown that TG/HDL can be used as an accurate and reliable tool to predict MetS [17, 21, 53]. Interestingly, the application of this ratio to predict CVD was proved in some studies [54, 55]. Insulin-resistant patients with TG/HDL ≥ 3.0 are diagnosed with MetS according to NCEP and IDF criteria [56]. In line with our findings, the AUC for TG/HDL was higher than other lipid ratios in predicting MetS [57, 58]. Thus, TG/HDL can be utilized as a diagnostic tool in addition to the other well-established diagnostic criteria for MetS.

Although WC is one of the main diagnostic criteria of MetS, it is not routinely measured during primary care visits [59]. It is well-established that WC, representing visceral lipid, is a health risk factor [60]. Recently, it was reported that WC is a better predictor of MetS than percentage body fat measured by impedance method among elderly [61].

We showed that in both men and women, the cutoff for non-HDL/HDL, TG/HDL, and LDL/HDL were 0.88 (0.85–0.90), 0.90 (0.88–0.93), and 0.87 (0.84–0.90), respectively. Hadaegh et al. revealed the optimal cutoff values for TG/HDL-C (4.7 in men and 3.7 in women) and TC/HDL (5.3 in both men and women) in order to predict the incidence of diabetes in men and women [62]. In another study, they also found that MetS in males with a TG/HDL-C level of 2.8–4.4 is five times greater than in those with a TG/HDL-C level of less than 2.8 [63].

Diagnostic criteria of MetS components might be different in various population. There is limited evidence of lipid ratios (i.e. TG/HDL, LDL/HDL, and non-HDL/HDL) for diagnosing MetS in the Iranian people. Given that early diagnosis of MetS might prevent severe complications, it is imperative to develop some practical criteria to distinguish healthy adults from those with MetS. Since the laboratory data for lipid profiles including TG and HDL are monitored routinely by health professionals in Iran, TG/HDL can play a pivotal role in screening MetS. However, it is worth mentioning that it should not be replaced with well-known criteria for MetS diagnosis.

### Strenghts and limitations

The first strength of our study is that it includes a large sample of elderly adults. Second, this study shows a primary care approach in geriatric medicine. The most important limitation of our study was that the bedridden elderly were not included in this study. Second, this is a cross sectional study, hence we recommend further prospective study to evaluate the association of lipid ratio with metablic syndrome. Third, since there was no information about sedentary life style of elderly people in our study, it was not included as cofounding variables. Fourth, because rural people were not included in this study, there was no information regarding the distribution of people based on rural and urban area.

## Conclusion

In conclusion, the results of this study showed a significant association between lipid ratios and metabolic syndrome among the elderly in Iran. Our results illustrated that the TG/HDL-C ratio is superior to the LDL-C/HDL-C ratio and the non-HDL/HDL ratio to predict metabolic syndrome among the elderly based on NCEP ATP III criteria.

Decleration.

## Data Availability

The datasets generated and/or analysed during the current study are not publicly available due to the privacy of participants but are available from the corresponding author on reasonable request.
